# Microsurgical Unroofing to Secure a Safe Corridor for C1 Screws in Bilateral Ponticulus Posticus: A Case Report

**DOI:** 10.7759/cureus.114517

**Published:** 2026-08-14

**Authors:** Kyota Kitagawa, Satoshi Maki, Takashi Hozumi, Seiji Ohtori

**Affiliations:** 1 Department of Orthopaedic Surgery, Graduate School of Medicine, Chiba University, Chiba, JPN; 2 Department of Orthopaedic Surgery, Chiba University Hospital, Chiba, JPN

**Keywords:** c1-c2 fixation, ponticulus posticus, spinal cord injury, spinal fusion surgery, vertebral artery (va)

## Abstract

Ponticulus posticus (PP) is a bony bridge arising from the posterior arch of C1 toward the superior articular process or lateral mass; when complete, it forms an arcuate foramen over the C1 vertebral artery (VA) groove and may convert the VA path into an osseous canal. This anatomy can narrow the usual corridor for C1 lateral mass screw (LMS) insertion. We report a 68-year-old man who sustained an upper cervical cord injury (ASIA Impairment Scale grade [AIS] B) after a fall. Computed tomography (CT) revealed continuous ossification of the posterior longitudinal ligament and anterior longitudinal ligament from C2 to T1, and magnetic resonance imaging showed canal stenosis due to a retro-odontoid pseudotumor with intramedullary signal change. Dynamic flexion radiograph demonstrated an atlas-dens interval of 5.6 mm and a space available for the spinal cord of 15.5 mm, while three-dimensional computed tomographic angiography identified bilateral complete PP with an aberrant VA course and no symptoms of vertebrobasilar insufficiency. After C1 laminectomy, the arcuate bony bridge was microsurgically unroofed with a 3-mm high-speed burr, enabling direct visualization and protection of the VA with neurosurgical sheets and a Penfield dissector; unroofing was complete on the left and partial on the right based on the bony bridge morphology and the VA exit point. An intraoperative navigation system confirmed screw entry points and trajectories, allowing placement of C1 screws. Immediate postoperative CT confirmed appropriate screw positions. Postoperative vascular imaging was not performed. At three months, the patient’s neurological status improved to AIS C, which indicates motor-incomplete spinal cord injury, with no implant-related complications. In bilateral complete PP, the risk of VA injury with C1 LMS has led to divergent recommendations ranging from avoidance to conditional use with protective techniques. Microsurgical unroofing placed the VA under direct visualization and protection, and navigation verified a controlled corridor, supporting intraoperative adjustments in complex C1-2 anatomy. In this setting, microsurgical unroofing with navigation permitted C1 LMS placement under direct VA visualization and protection and may be a reasonable option when a controlled corridor can be created in selected patients.

## Introduction

Ponticulus posticus (PP) is a bony bridge extending from the posterior arch of the atlas (C1) to the superior articular process or lateral mass, over the groove where the vertebral artery (VA) courses along the cranial surface of the posterior arch before entering the foramen magnum [1]. In complete PP, this bridge forms an arcuate foramen that can enclose the VA and make the posterior arch appear broader on imaging. Reported prevalence varies widely [2,3], and complete bilateral forms can substantially constrain the lateral mass screw (LMS) trajectory because both potential C1 screw corridors may be close to an osseous VA canal [4]. Secure C1-2 fixation is important for restoring stability and preventing further spinal cord injury in selected cases of atlantoaxial instability. Because VA injury is a feared complication of C1 screws, some authors consider PP a relative contraindication to LMS in some settings, whereas others describe techniques that enable insertion with protective measures under selected conditions [5-7]. Alternative strategies when C1 LMS placement is considered risky include C1 posterior arch screws, C1 laminar hook constructs, C1-2 transarticular screws, and occipitocervical fusion. We report a trauma case with bilateral complete PP in which microsurgical unroofing of the arcuate foramen combined with navigation allowed long-trajectory C1 LMS fixation under direct VA visualization and protection. We outline decision points, technical steps, and short-term outcomes, and we discuss how this strategy compares with alternatives, as described by Tan et al. [8].

## Case presentation

**Figure 1:**
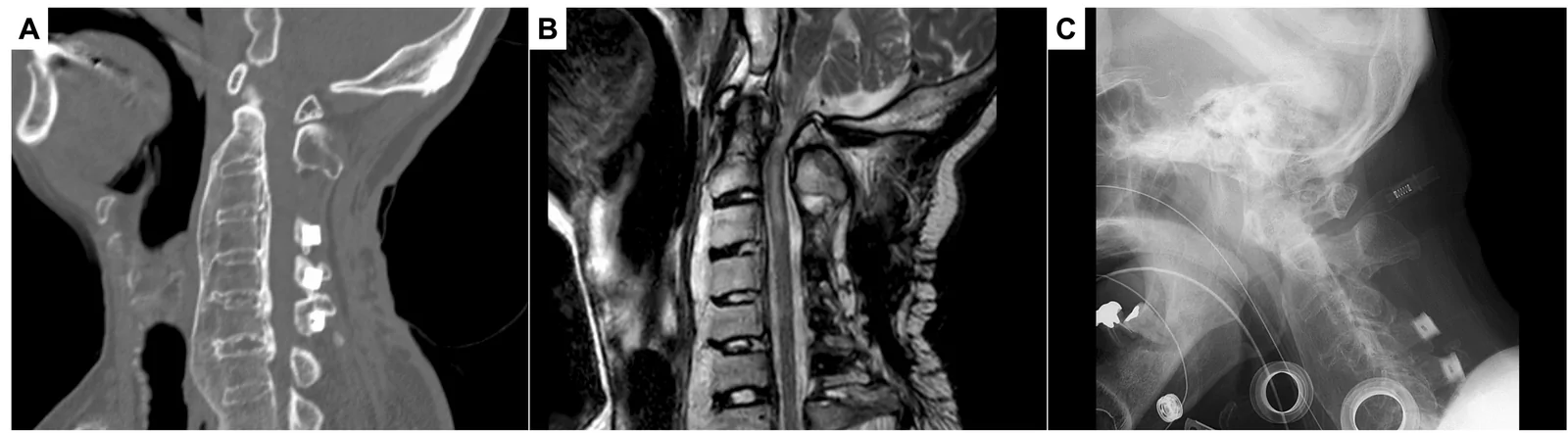
Preoperative CT, MRI, and dynamic imaging of the cervical spine. Mid-sagittal CT (A) demonstrates continuous OALL and OPLL from C2 to T1 without apparent acute injuries such as fracture, disc injury, or posterior element injury. Mid-sagittal T2-weighted MRI (B) shows canal stenosis due to a retro-odontoid pseudotumor accompanied by intramedullary signal change from C1 to C3. Dynamic imaging in neck flexion (C) reveals an ADI of 5.6 mm and a SAC of 15.5 mm. Abbreviations: ADI, atlas-dens interval; CT, computed tomography; MRI, magnetic resonance imaging; OALL, ossification of the anterior longitudinal ligament; OPLL, ossification of the posterior longitudinal ligament; SAC, space available for the spinal cord.

A 68-year-old man presented with an upper cervical spinal cord injury after a fall on the stairs and was classified as ASIA Impairment Scale (AIS) grade B. Because of respiratory insufficiency after the injury, he required ventilatory support. The patient’s key muscles below C5 were flaccid bilaterally, but pinprick sensation was slightly preserved in the upper extremities and sacral segments. The patient’s medical history included French-door laminoplasty from C3 to C6. Computed tomography (CT) demonstrated continuous ossification of the posterior longitudinal ligament and ossification of the anterior longitudinal ligament from C2 to T1 with no acute disc or posterior element injury (Figure 1A). 

**Figure 2:**
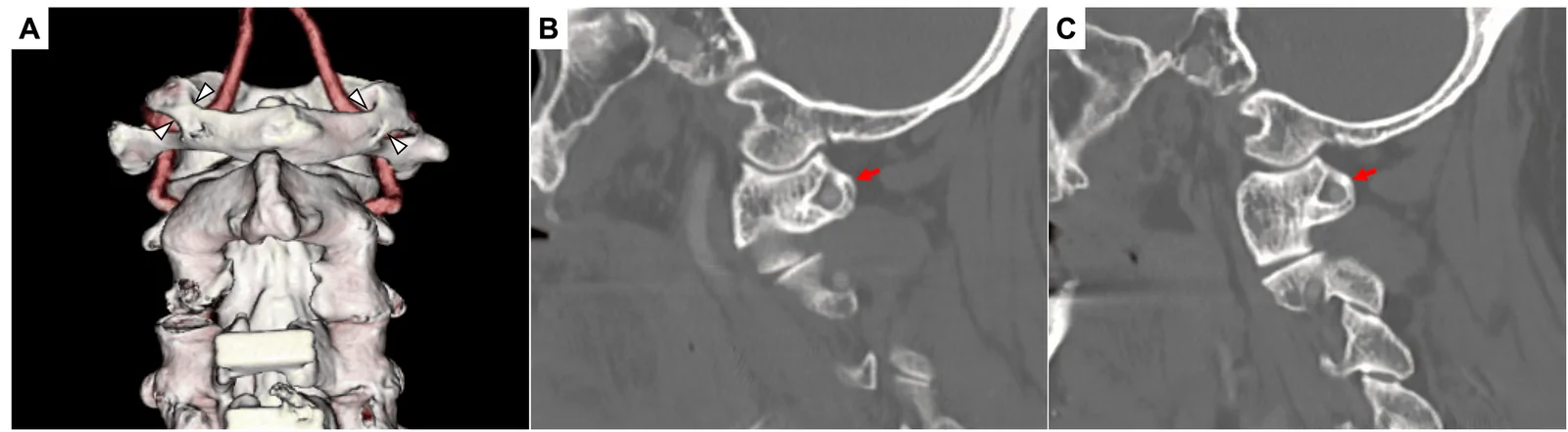
Three-dimensional CT angiography of bilateral complete ponticulus posticus. Posterior three-dimensional CT angiographic view (A) shows bilateral complete PP. The posterior arch appears broader because of bony bridges (white arrowheads) connecting the posterior arch to the cranial part of the lateral mass over the vertebral artery (VA). Parasagittal views of the preoperative CT scan (B, right side; C, left side) demonstrate a complete bony roof (red arrows) extending from the posterior arch to the lateral mass and surrounding the VA. Abbreviations: CT, computed tomography; PP, ponticulus posticus; VA, vertebral artery.

Magnetic resonance imaging showed canal stenosis due to a retro-odontoid pseudotumor with intramedullary hyperintensity (Figure 1B). A dynamic flexion radiograph revealed an atlas-dens interval of 5.6 mm and a space available for the spinal cord of 15.5 mm (Figure 1C). Three-dimensional computed tomographic angiography revealed bilateral complete PP with an anomalous VA course (Figure 2). The bony bridges roofed the VA groove bilaterally, and the broader right-sided bridge overlapped the planned C1 LMS corridor. We therefore planned direct VA exposure and protection before screw insertion. 

Notably, the patient had no history of vertebrobasilar insufficiency symptoms.

Given the ankylosed subaxial cervical spine (C2-T1) and instability at the adjacent C1-C2 level, a rigid C1-C2 construct was considered appropriate. We therefore planned the longest feasible C1 LMS despite the bilateral complete PP.

**Figure 3:**
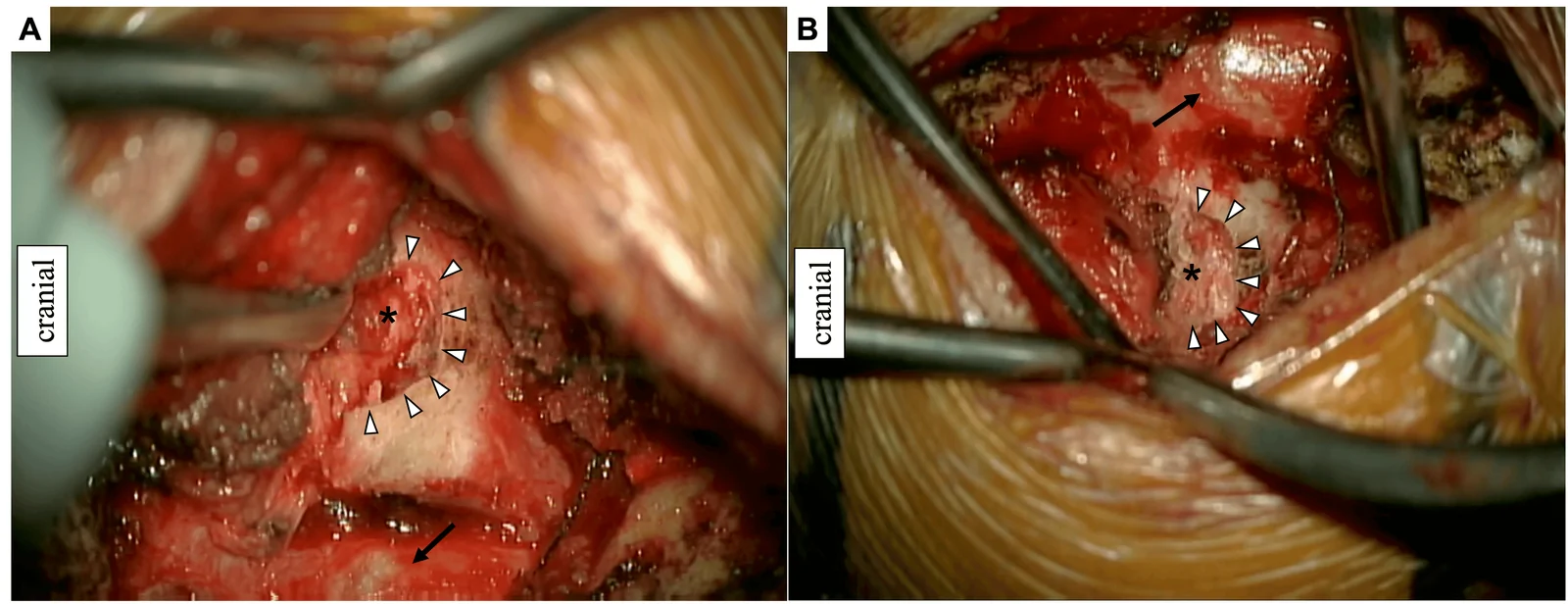
Intraoperative findings during microsurgical unroofing. Intraoperative photographs from the right (A) and left (B) sides after unroofing are shown. Black arrows indicate the dura mater, asterisks mark the exposed VA, and white arrowheads indicate the resection margins of the arcuate bony bridge. The images demonstrate the created corridor for C1 screw placement; after unroofing, a Penfield dissector can be interposed between the VA and posterior arch to protect the artery. Abbreviations: VA, vertebral artery.

During surgery, the C2 nerve root was not specifically manipulated, and C1 laminectomy was performed. The bony bridge was evaluated first, including its width, lateral edge, and relationship to the planned screw corridor. The unroofing procedure was performed as follows: (1) on the left side, where complete unroofing was planned, bone removal was started at the lateral edge of the bony bridge; (2) a high-speed burr was used to remove bone gradually in small amounts, leaving the residual cortex thin enough for the deeper layer to become translucent rather than removing the roof en bloc; (3) the remaining thin cortex was opened with a Penfield dissector; (4) the VA was protected with neurosurgical sheets and a Penfield dissector, and subsequent drilling was performed over these protective materials; and (5) bone removal on the left side was connected from the lateral edge of the bony bridge to the superior edge of the C1 posterior arch to complete unroofing. On the right side, where partial unroofing was planned, bone removal was started from the medial side and extended only as needed to allow interposition of the protective materials between the VA and posterior arch. The arcuate bony bridge was microsurgically unroofed to expose the VA for direct visualization and protection, demonstrating the created corridor for C1 screw placement (Figure 3A, 3B). 

Unroofing was complete on the left and partial on the right. Because partial unroofing on the right provided adequate space to interpose protective materials between the VA and posterior arch, and navigation confirmed an acceptable screw trajectory, we considered additional right-sided unroofing unnecessary (Figure 3B). Intraoperative navigation guided the screw entry points and confirmed the trajectories after one O-arm scan. C1 LMS and C2 pedicle screws were placed routinely.

**Figure 4:**
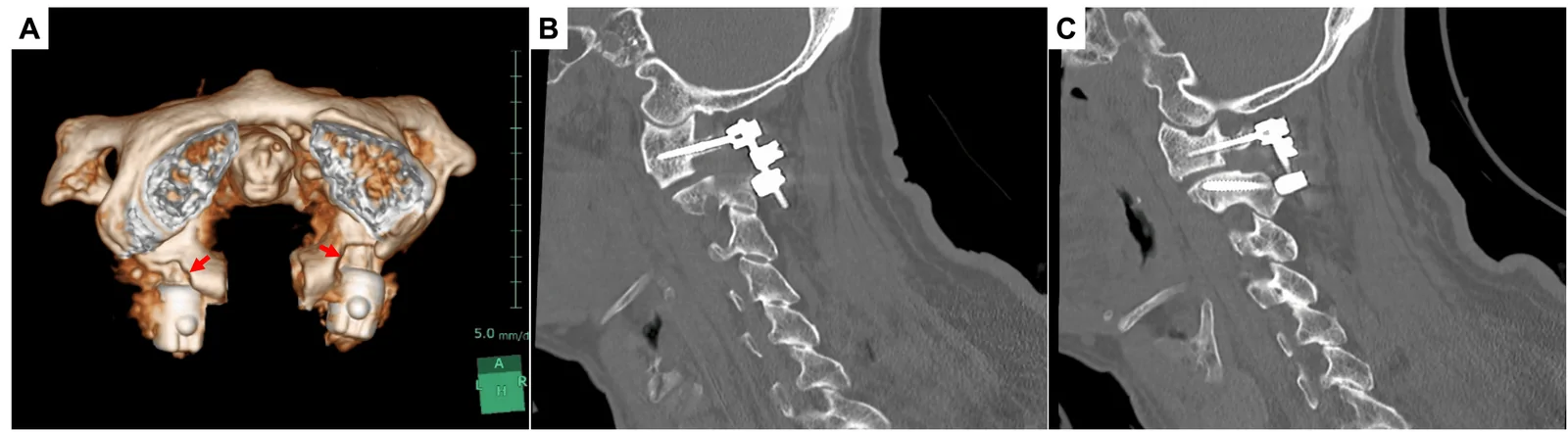
Postoperative CT demonstrating screw placement and the unroofing. The cranial aspects of the posterior arch are exposed by unroofing (A, red arrows). C1 LMSs are correctly placed (B, right side; C, left side). Abbreviations: CT, computed tomography; LMS, lateral mass screw.

The operative time was 4 hours 38 minutes, and the estimated blood loss was 200 mL. Postoperative CT confirmed appropriate screw positions (Figure 4). Postoperative vascular imaging was not performed. At 3-month follow-up, the patient’s neurological status improved to AIS C. According to the ASIA/ISCoS worksheet, AIS C is motor-incomplete spinal cord injury in which “less than half of key muscle functions below the single neurological level of injury have a muscle grade ≥ 3” [8]. No implant-related complications, vertebrobasilar symptoms, postoperative occipital neuralgia, or C2-distribution numbness occurred, and 3-month CT suggested early fusion.

## Discussion

Complete PP forms a rigid osseous canal around the VA, limiting direct access to the artery and thereby narrowing the usual corridor for C1 LMS placement. Therefore, the use of C1 LMS in patients with PP remains controversial because of the risk of VA injury [1,4,5,10], and only a few reports have described surgical techniques for inserting C1 LMS in the presence of complete PP [5-7]. Hattori et al. reported C1 screw insertion from the caudal side of the C2 nerve root [5]. Fujisawa et al. used a C1 laminar hook on the posterior arch instead of a C1 LMS [7]. In our case, microsurgical unroofing created a controlled corridor that placed the VA under direct visualization and protection with neurosurgical sheets and a Penfield dissector, allowing a long-trajectory C1 LMS while maintaining intraoperative vascular control. The procedure differed by side: complete unroofing on the left was performed from the lateral edge of the bony bridge, whereas partial unroofing on the right was performed from the medial side until sufficient space for VA protection was obtained. This unroofing technique has rarely been discussed specifically for facilitating C1 screw placement. A report by Jung et al. described the effectiveness of unroofing when a C1 screw is required in patients with complete PP [10]. Although unroofing is still uncommon in the context of instrumentation, there are several neurosurgical reports of decompression surgery to treat vertebral artery tethering symptoms due to complete PP [11,12]. In addition to unroofing, we used O-arm navigation to verify entry points and trajectories, supporting intraoperative adjustments and confirming placement in complex C1-2 anatomy [13,14]. When a controlled environment for the VA cannot be achieved or when a safe long-trajectory C1 LMS corridor does not exist, alternative constructs, such as C1 posterior arch screws, C1 laminar hook constructs, or C1-2 transarticular screws, should be considered based on case-specific anatomy and VA risk [16-18]. Occipitocervical fusion may also be an option, although it requires a more cranially extended upper instrumented level and can further limit postoperative range of motion.

In our case, the C1-2 segment showed moderate instability secondary to the retro-odontoid pseudotumor with the subaxial cervical spine ankylosed from C2 to T1 at the adjacent level. In this anatomical and biomechanical context, we prioritized a long-trajectory screw through the posterior arch and lateral mass [19] to maximize cortical purchase and improve construct stiffness [19]. This rationale supports considering unroofing to enable LMS rather than defaulting to shorter or fewer anchors when a controlled corridor for VA protection can be established.

This report has several limitations. It describes a single patient, and follow-up was limited to 3 months. Postoperative vascular imaging was not performed; therefore, postoperative VA patency was not objectively confirmed. Accordingly, our conclusions are limited to the observed short-term clinical and radiographic outcome, and further cases with longer follow-up are needed to clarify the safety, reproducibility, and indications of this technique.

## Conclusions

In this case of bilateral complete ponticulus posticus, microsurgical unroofing combined with navigation allowed C1 lateral mass screw placement under direct visualization and protection of the vertebral artery. The patient had no observed implant-related complication, vertebrobasilar symptoms, postoperative occipital neuralgia, or C2-distribution numbness during the 3-month follow-up, and CT suggested early fusion. This technique may be considered in selected patients when preoperative imaging and intraoperative findings indicate that a controlled corridor for vertebral artery protection can be established. Further cases and longer follow-up are needed to clarify its safety, reproducibility, and indications.

## References

[1] Tubbs RS, Johnson PC, Shoja MM, Loukas M, Oakes WJ (2007). Foramen arcuale: anatomical study and review of the literature. J Neurosurg Spine.

[2] Elliott RE, Tanweer O (2014). The prevalence of the ponticulus posticus (arcuate foramen) and its importance in the Goel-Harms procedure: meta-analysis and review of the literature. World Neurosurg.

[3] Sekerci AE, Soylu E, Arikan MP, Ozcan G, Amuk M, Kocoglu F (2015). Prevalence and morphologic characteristics of ponticulus posticus: analysis using cone-beam computed tomography. J Chiropr Med.

[4] Young JP, Young PH, Ackermann MJ, Anderson PA, Riew KD (2005). The ponticulus posticus: implications for screw insertion into the first cervical lateral mass. J Bone Joint Surg Am.

[5] Hattori S, Wada K, Watanabe F, Matsutani S (2024). C1 lateral mass screw insertion caudally from the C2 nerve root to avoid craniocervical fusion in a patient with atlantoaxial subluxation associated with ponticulus posticus: a case report. Cureus.

[6] Zhang XL, Huang DG, Wang XD, Zhu JW, Li YB, He BR, Hao DJ (2017). The feasibility of inserting a C1 pedicle screw in patients with ponticulus posticus: a retrospective analysis of eleven patients. Eur Spine J.

[7] Fujisawa M, Wakahara S, Inamasu J (2025). Unstable upper cervical spine injury with concomitant bilateral ponticulus posticus: a case report. Asian J Neurosurg.

[8] Tan M, Wang H, Wang Y (2003). Morphometric evaluation of screw fixation in atlas via posterior arch and lateral mass. Spine (Phila Pa 1976).

[9] Kirshblum SC, Burns SP, Biering-Sorensen F (2026). American Spinal Injury Association: International Standards for
Neurological Classification of Spinal Cord Injury (ISNCSCI) Worksheet. J Spinal Cord Med.

[10] Arslan D, Ozer MA, Govsa F, Kıtıs O (2018). The ponticulus posticus as risk factor for screw insertion into the first cervical lateral mass. World Neurosurg.

[11] Jung YG, Lee BJ, Park W, Park JH (2021). C1-2 pedicle screw fixation for ponticulus posticus and duplication of vertebral artery: 2-dimensional operative video. Oper Neurosurg.

[12] Lukianchikov V, Lvov I, Grin A, Kordonskiy A, Polunina N, Krylov V (2018). Minimally invasive surgical treatment for vertebral artery compression in a patient with one-sided ponticulus posticus and ponticulus lateralis. World Neurosurg.

[13] Yakel S, Nussbaum ES, Patel PD (2021). Surgical decompression of the vertebral artery in a patient with ponticulus posticus: a case report. SN Compr Clin Med.

[14] Smith JD, Jack MM, Harn NR, Bertsch JR, Arnold PM (2016). Screw placement accuracy and outcomes following O-arm-navigated atlantoaxial fusion: a feasibility study. Global Spine J.

[15] Hur JW, Kim JS, Ryu KS, Shin MH (2019). Accuracy and safety in screw placement in the high cervical spine: retrospective analysis of O-arm-based navigation-assisted C1 lateral mass and C2 pedicle screws. Clin Spine Surg.

[16] Nagoshi N, Suda K, Morita T (2014). C1 posterior arch screw as an auxiliary anchor in posterior reconstruction for atlantoaxial dislocation associated with type II odontoid fracture: a case report and review of the literature. Springerplus.

[17] Guo X, Ni B, Zhao W, Wang M, Zhou F, Li S, Ren Z (2009). Biomechanical assessment of bilateral C1 laminar hook and C1-2 transarticular screws and bone graft for atlantoaxial instability. J Spinal Disord Tech.

[18] Liu C, Kamara A, Yan Y (2020). Biomechanical study of C1 posterior arch crossing screw and C2 lamina screw fixations for atlantoaxial joint instability. J Orthop Surg Res.

[19] Zindrick MR, Wiltse LL, Widell EH, Thomas JC, Holland WR, Field BT, Spencer CW (1986). A biomechanical study of intrapeduncular screw fixation in the lumbosacral spine. Clin Orthop Relat Res.

